# Author Correction: Integrating social vulnerability into high-resolution global flood risk mapping

**DOI:** 10.1038/s41467-025-56676-2

**Published:** 2025-02-07

**Authors:** Sean Fox, Felix Agyemang, Laurence Hawker, Jeffrey Neal

**Affiliations:** 1 School of Geographical Sciences & Cabot Institute, University of Bristol, Bristol, UK; 2 Department of Planning, Property & Environmental Management, University of Manchester, Manchester, UK; 3 Fathom, Bristol, UK

Correction to: *Nature Communications* 10.1038/s41467-024-47394-2, published online 11 April 2024

The original version of this Article contained an error in the in-text references to the figures. The correct version includes the correct in-text references. This has been corrected in both the PDF and HTML versions of the Article.

