# Supplementary material for: Integrating social vulnerability into high-resolution global flood risk mapping
Source: Nat Commun. 2024 Apr 11;15:3155. doi: 10.1038/s41467-024-47394-2 (PMC11009285; doi:10.1038/s41467-024-47394-2)
Supplement: Supplementary file 1 — Supplementary Information [file 41467_2024_47394_MOESM1_ESM.pdf]

SUPPLEMENTARY INFORMATION

Global sensitivity analysis with multidimensional relative poverty data

Supplementary Figure 1 reproduces Figure 2b from the main text with an alternative proxy for social vulnerability: the Global Gridded Relative Deprivation Index from the Center for International Earth Science Observation at Columbia University. The broad trends are similar, although the overall estimates of the number of people at risk is much smaller. This is due to much more limited geographic coverage, including in many populated flood-prone regions. While the GDP data contain over 200 million cells, the multidimensional deprivation index contains just 22 million—just 11% of the total available in the GDP data.

Figure S1. Comparison of global and regional estimates of flood risk estimates with multidimensional relative poverty data.

	Population by EPE Risk Score (millions)					Population by VARI Score (millions)				
	1	*2*	*3*	*4*	*5*	*1*	*2*	*3*	*4*	*5*
Africa	8	23	27	35	71	2	39	65	38	20
Americas	7	16	17	20	62	13	62	32	11	3
Asia	66	160	187	208	412	53	366	316	238	60
Europe	16	21	20	23	35	27	69	16	2	0
Oceania	0	0	0	0	1	0	1	0	0	1
Total	98	219	251	286	580	96	536	429	290	83

Figure S2. Cell-level correlation between GDP per capita & Relative deprivation

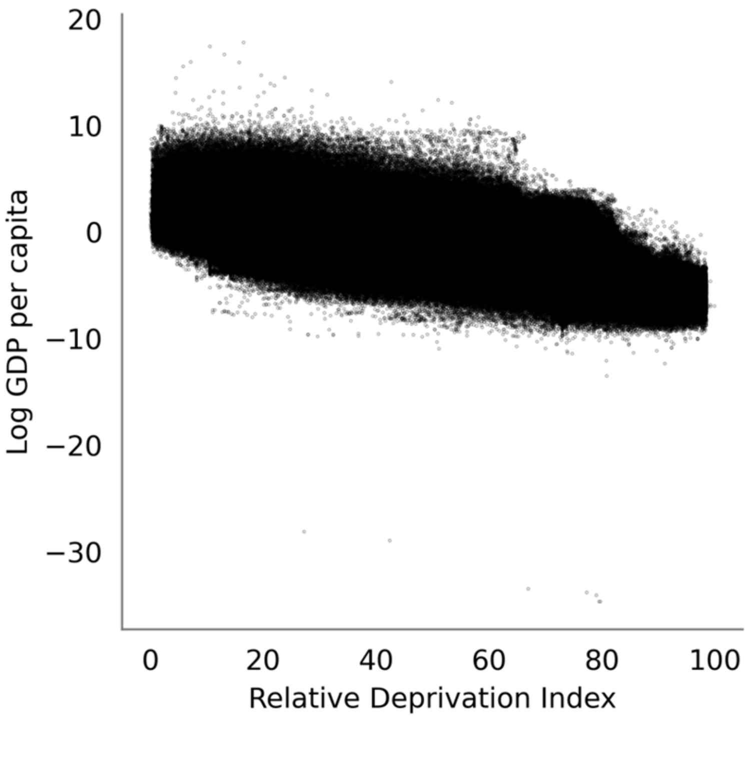

However, there is clear cell-level correlation between these variables. Supplementary Figure 2 shows the cell-level bivariate correlation between the log of GDP per capita and multidimensional relative deprivation from a sample of 10% of the cells from all countries in our sample (*n*=4,868,628). Sampling was used for computational efficiency and all cells with GDP per capita values of 0 were dropped. There is a clear negative correlation between relative deprivation and GDP per capita (r = -0.63; R-squared = 0.40). Given this strong cell-level correspondence between these data, the GDP data are preferable due to much greater geographic coverage.

Country-level sensitivity analysis with alternative flood depth thresholds

Supplementary Figure 3 shows (i) the total number of people exposed to a flood hazard across our return period spectrum (up to 1/1000), and (ii) the total number of people that fall into the highest risk category (5) of expected population exposure (EPE) and our vulnerability-adjusted risk index (VARI) for Nigeria, Pakistan, and Vietnam at three depth thresholds: 10cm, 50cm and 100cm.

Figure S3. Population at risk at variable flood depth thresholds

		EPE population (000s)		VARI Flood pop. (000s)	
		Risk Score 5	Total	Risk Score 5	Total
Nigeria	10cm	7712	20812	3168	20812
	50cm	4794	12957	1837	12957
	100cm	3144	8610	1098	8610
Pakistan	10cm	27355	105804	9998	105804
	50cm	17810	70189	7188	70189
	100cm	12589	48370	5603	48370
Vietnam	10cm	14358	47518	3955	47518
	50cm	11388	40317	3263	40317
	100cm	9928	35023	9928	35023

Overall, the higher the flood depth threshold, the lower the estimated population at risk across both measures in all three countries, particularly in the highest risk category. We also find a similar shift in the distribution across risk scores when the whole population distribution is considered, as illustrated in Supplementary Figures 4-6. In sum, increasing the flood depth threshold reduces the estimated population-at-risk using both approaches, but it has little effect on how our understanding of the distribution of risk changes when a proxy for social vulnerability is incorporated into flood risk assessment.

(a)

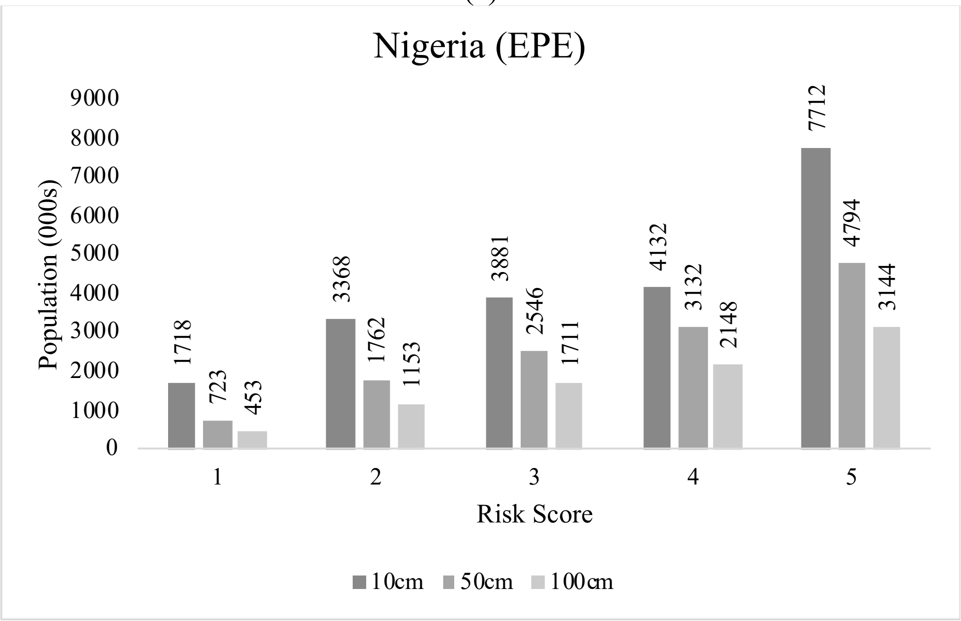

(b)

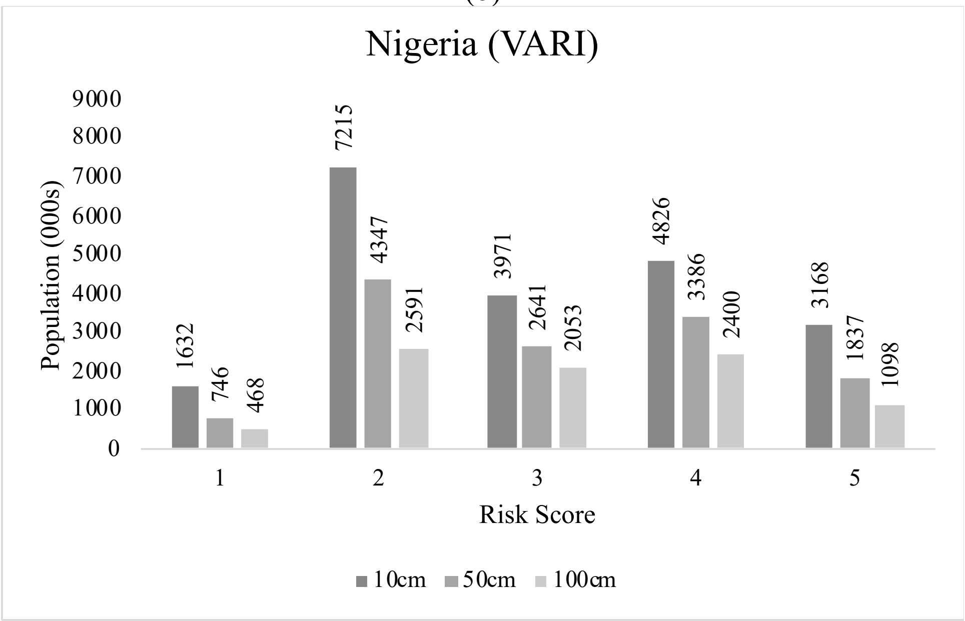

Figure S4. Population at risk in Nigeria by Risk Score at 10cm, 50cm & 100cm flood depth thresholds. S4a shows estimates of the number of people by flood Risk Score using expected population exposure (EPE) alone; S4b shows estimates with the vulnerability adjusted risk index (VARI).

(a)

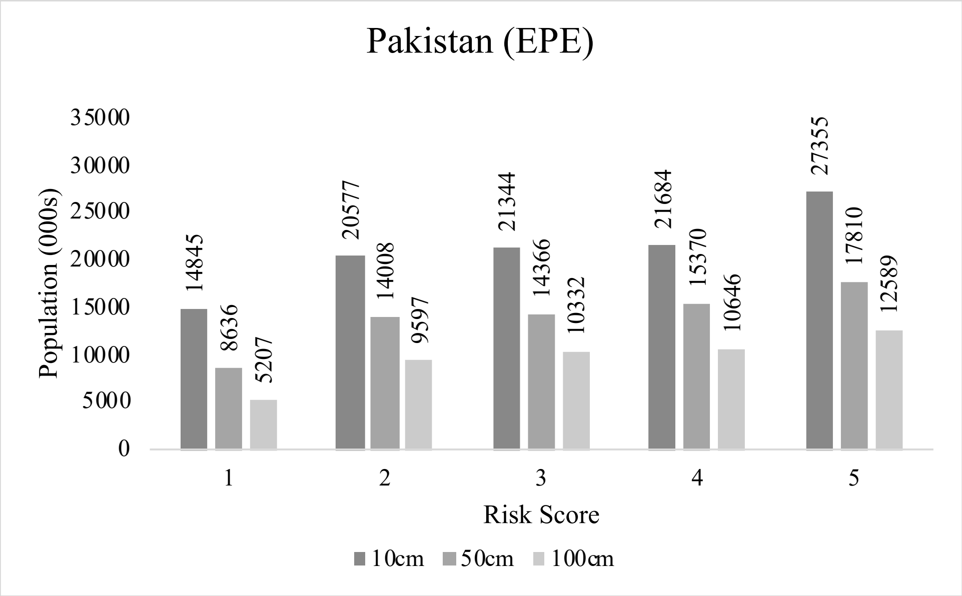

(b)

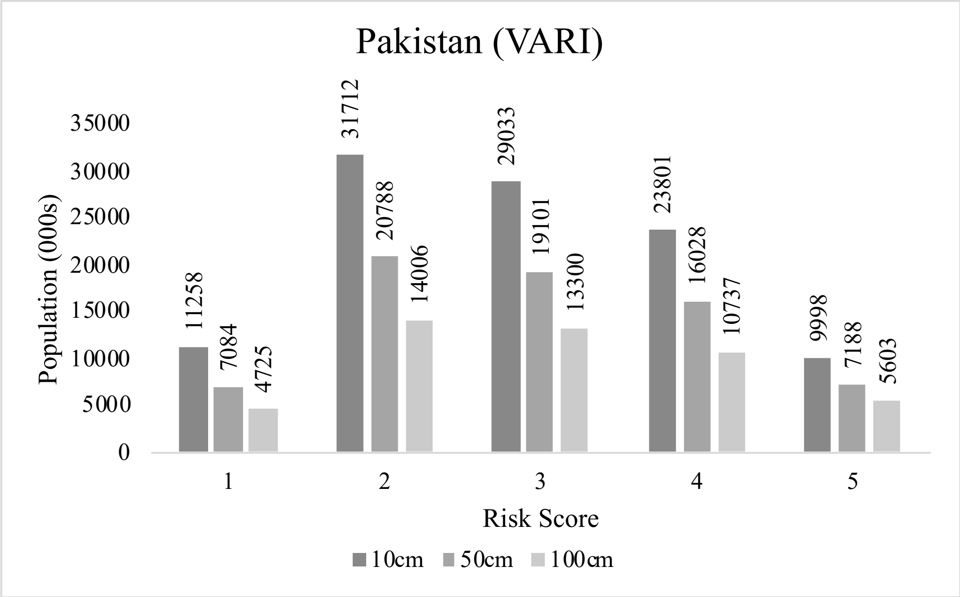

Figure S5. Population at risk in Pakistan by Risk Score at 10cm, 50cm & 100cm flood depth thresholds. S5a shows estimates of the number of people by flood Risk Score using expected population exposure (EPE) alone; S5b shows estimates with the vulnerability adjusted risk index (VARI).

(a)

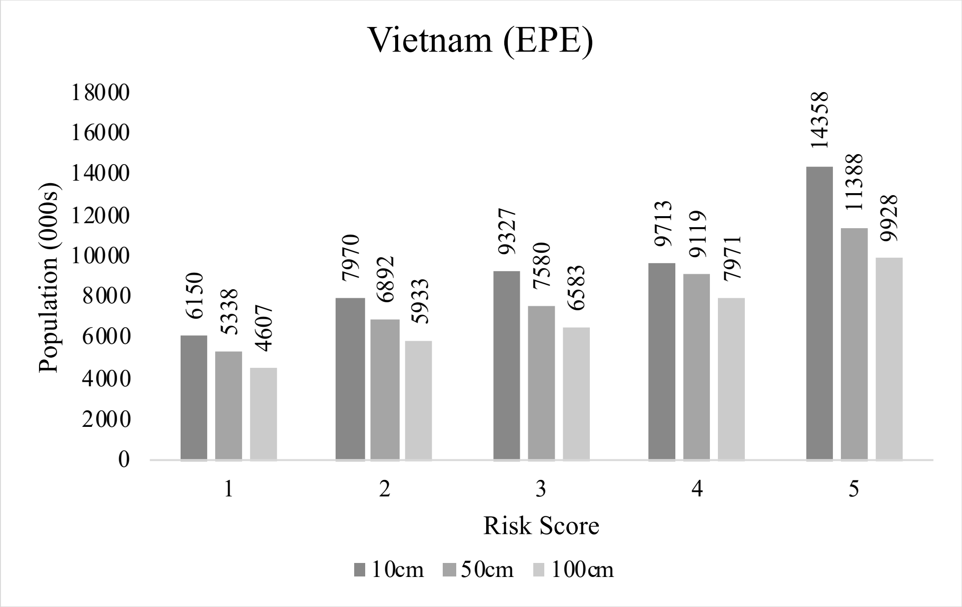

(b)

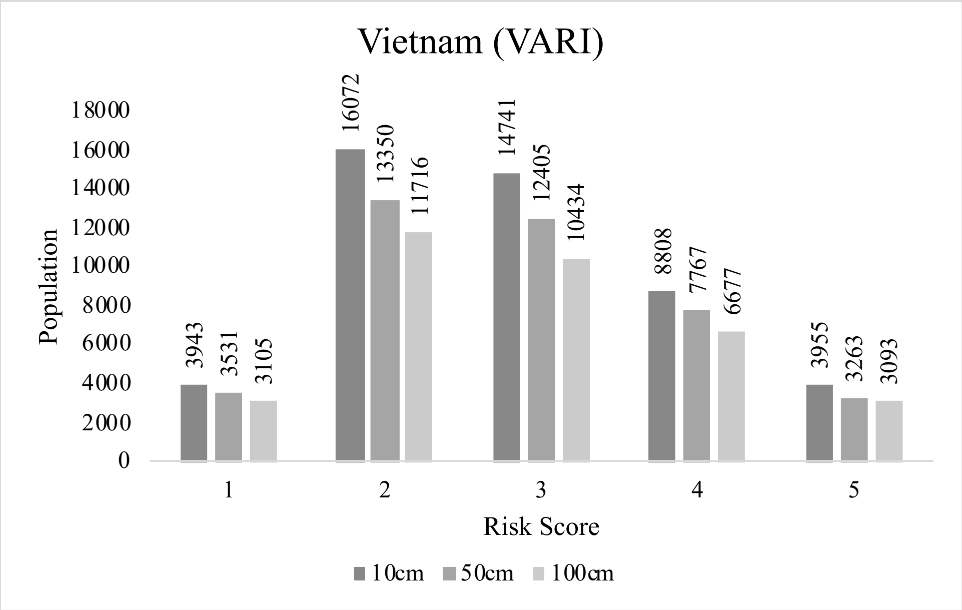

Figure S6. Population at risk in Vietnam by Risk Score at 10cm, 50cm & 100cm flood depth thresholds. S6a shows estimates of the number of people by flood Risk Score using expected population exposure (EPE) alone; S6b shows estimates with the vulnerability adjusted risk index (VARI).
